# Augmented brain function by coordinated reset stimulation with slowly varying sequences

**DOI:** 10.3389/fnsys.2015.00049

**Published:** 2015-03-31

**Authors:** Magteld Zeitler, Peter A. Tass

**Affiliations:** ^1^Research Center Jülich, Institute of Neuroscience and Medicine, Neuromodulation (INM-7)Jülich, Germany; ^2^Department of Neurosurgery, Stanford UniversityStanford, CA, USA; ^3^Department of Neuromodulation, University of CologneCologne, Germany

**Keywords:** coordinated reset, slowly varying sequences, desynchronization, spike timing-dependent plasticity, anti-kindling

## Abstract

Several brain disorders are characterized by abnormally strong neuronal synchrony. Coordinated Reset (CR) stimulation was developed to selectively counteract abnormal neuronal synchrony by desynchronization. For this, phase resetting stimuli are delivered to different subpopulations in a timely coordinated way. In neural networks with spike timing-dependent plasticity CR stimulation may eventually lead to an anti-kindling, i.e., an unlearning of abnormal synaptic connectivity and abnormal synchrony. The spatiotemporal sequence by which all stimulation sites are stimulated exactly once is called the stimulation site sequence, or briefly sequence. So far, in simulations, pre-clinical and clinical applications CR was applied either with fixed sequences or rapidly varying sequences (RVS). In this computational study we show that appropriate repetition of the sequence with occasional random switching to the next sequence may significantly improve the anti-kindling effect of CR. To this end, a sequence is applied many times before randomly switching to the next sequence. This new method is called SVS CR stimulation, i.e., CR with slowly varying sequences. In a neuronal network with strong short-range excitatory and weak long-range inhibitory dynamic couplings SVS CR stimulation turns out to be superior to CR stimulation with fixed sequences or RVS.

## Introduction

Abnormally strong neuronal synchronization characterizes several brain disorders, e.g., Parkinson's disease (Lenz et al., 1994; Nini et al., 1995; Hammond et al., 2007), epilepsy (Wong et al., 1986; Schomer and Lopes da Silva, 2010), and tinnitus (Ochi and Eggermont, 1997; Llinas et al., 1999; Weisz et al., 2005; Eggermont and Tass, 2015). Coordinated reset (CR) stimulation (Tass, 2003a,b) was developed in order to specifically counteract abnormal neuronal synchrony by desynchronization (Tass, 1999). CR stimulation means to deliver phase resetting stimuli at different times to different sub-populations involved in abnormal neuronal synchronization (Tass, 2003a,b). Computational studies showed that in neuronal populations with spike timing-dependent plasticity (STDP) (Gerstner et al., 1996; Markram et al., 1997; Bi and Poo, 1998; Feldman, 2000) CR stimulation has long-lasting, sustained effects (Tass and Majtanik, 2006; Hauptmann and Tass, 2007; Popovych and Tass, 2012). This is because CR stimulation employs the multistability of neuronal networks with STDP (Tass and Majtanik, 2006; Hauptmann and Tass, 2007; Maistrenko et al., 2007; Popovych and Tass, 2012). CR-stimulation causes a desynchronization and in turn, due to STDP (Gerstner et al., 1996; Markram et al., 1997; Bi and Poo, 1998; Feldman, 2000), leads to a decrease of the mean synaptic weight. In this way, CR stimulation shifts the neuronal network from a pathological attractor with up-regulated synchrony and connectivity to a physiological attractor with down-regulated synchrony and connectivity (Tass and Majtanik, 2006; Hauptmann and Tass, 2007; Popovych and Tass, 2012). In this way CR applied induces an unlearning of the abnormal synaptic connectivity and abnormal neuronal synchrony, so that an anti-kindling is achieved (Tass and Majtanik, 2006).

Computational studies showed that anti-kindling can robustly be achieved in networks of spiking or bursting model neurons where the neurons interact via plastic excitatory and inhibitory synapses (Popovych and Tass, 2012; Tass and Popovych, 2012). These studies show also that anti-kindling occurs irrespective of whether CR stimulation is delivered to the somata or to excitatory or inhibitory synapses.

In accordance with these computational findings, long-lasting CR-induced desynchronization was achieved in pre-clinical as well as clinical studies with invasive and non-invasive stimulation modalities. Electrical CR stimulation induced long-lasting desynchronization in rat hippocampal slice rendered epileptic by magnesium withdrawal (Tass et al., 2009). Therapeutic long-lasting after-effects of electrical CR deep brain stimulation were observed in parkinsonian non-human primates (Tass et al., 2012b). Unilateral CR stimulation applied to the subthalamic nucleus (STN) of parkinsonian MPTP monkeys for only 2 h per day during 5 subsequent days caused significant sustained bilateral therapeutic after-effects for at least 30 days, while no after-effects were induced by standard permanent high-frequency deep brain stimulation (Tass et al., 2012b). By the same token, lasting after-effects of electrical CR stimulation of the STN were also verified in parkinsonian patients (Adamchic et al., 2014a). So far, non-invasive CR stimulation was realized with acoustic stimuli and applied to the treatment of chronic subjective tinnitus (Tass and Popovych, 2012; Tass et al., 2012a). In a proof of concept-study it was shown that acoustic CR stimulation causes a statistically and clinically significant and sustained reduction of tinnitus symptoms (Adamchic et al., 2012a,b; Tass et al., 2012a) along with a concomitant reduction of abnormal neuronal synchrony (Tass et al., 2012a; Adamchic et al., 2014b), abnormal effective connectivity (Silchenko et al., 2013) and abnormal cross-frequency coupling (Adamchic et al., 2014c) within a tinnitus-related network of brain areas.

We here set out to further improve the efficacy of CR stimulation by focusing on a key element of CR, the stimulation site sequence, i.e., the temporal sequence of activating the different stimulation sites exactly once, which in what follows will briefly be called *sequence*. Keeping the sequence constant for all stimulation cycles is optimal in neuronal networks without STDP, since it enables optimal desynchronization at minimal intensities (Tass, 2003a,b). The situation gets more sophisticated in the presence of STDP. In a network of phase oscillators with couplings subject to STDP the sequence was randomly varied from cycle to cycle in order to avoid reverberations which might possibly lead to the formation of sequence-related neuronal subclusters and/or to a delayed anti-kindling (Tass and Majtanik, 2006). However, in several computational studies addressing different aspects of CR a robust anti-kindling was achieved with CR stimulation with fixed sequence (Hauptmann and Tass, 2007, 2009; Tass and Hauptmann, 2007, 2009) as well as with sequences randomly varying form cycle to cycle (Tass and Majtanik, 2006; Tass and Hauptmann, 2006; Popovych and Tass, 2012; Tass and Popovych, 2012; Ebert et al., 2014). We denote CR stimulation with sequences randomly varied from cycle to cycle as *RVS CR stimulation*, i.e., CR with rapidly varying sequences, whereas CR stimulation with fixed sequence is called *FS CR stimulation*, i.e., CR stimulation with fixed sequence. Although some findings indicated that RVS CR might lead to a quicker anti-kindling (Tass and Majtanik, 2006), so far no systematic comparison or deeper analysis was performed. In pre-clinical and clinical studies mainly RVS CR stimulation was applied (Tass et al., 2012a,b; Adamchic et al., 2014c), while FS CR stimulation was used only in an *in vitro* experiment (Tass et al., 2009). The available results do not allow to judge whether RVS CR or FS CR stimulation or possibly another variant of CR might be superior.

In this study we investigate the efficacy of a new CR stimulation variant for which a sequence is repeated during *n* stimulation cycles in a row before randomly switching to the next sequence. This type of CR will be called *SVS-n CR stimulation*, where SVS stands for slowly varying sequences. We show that repetition with occasionally switching of the sequence may significantly improve the performance of CR stimulation, leading to a more robust and quicker anti-kindling. To this end, we use a neuronal network model with STDP as described in Section Materials and Methods. The impact of the RVS and the SVS-n CR stimulation are compared in Section Slowly Varying Sequences Boost CR Stimulation Effect. Finally, in Section Optimal Number of Different Sequences Used for SVS CR Stimulation we demonstrate that optimal anti-kindling requires both variation and substantial repetition of the sequence. In fact, a sequence has to be repeated sufficiently often, e.g., at least 25 times, before randomly switching to another sequence.

## Materials and methods

### Conductance-based hodgkin-huxley model

The neural network used in this study consisted of *N* (*N* = 200) spiking conductance-based Hodgkin-Huxley neurons (Hodgkin and Huxley, 1952). The membrane potential *V* of each neuron *i* (*i* = 1, …, *N*) is characterized by Hansel et al. (1993), Popovych and Tass (2012):

(1) $$\begin{array}{l} C\frac{dV_{i}}{dt}=I_{i}-g_{Na}m_{i}^{3}h_{i}\left(V_{i}-V_{Na}\right)-g_{K}n_{i}^{4}\left(V_{i}-V_{K}\right)\\ \;-\;g_{l}\left(V_{i}-V_{l}\right)+S_{i}+F_{i}. \end{array}$$

*C* is the membrane capacitance, *I*_*i*_ the constant depolarizing current injected into neuron *i*, *S*_*i*_ is the current that represents synaptic input of the neurons within the network to neuron *i* and *F*_*i*_ is the current induced in neuron *i* by CR stimulation. Values used in this study are: *C* = 1 μF/cm^2^, maximum conductance per unit area for the sodium, potassium and leak currents, *g*_*Na*_ = 120 mS/cm^2^, *g*_*K*_ = 36 mS/cm^2^, *g*_*l*_ = 0.3 mS/cm^2^, with sodium reversal potential *V*_*Na*_ = 50 mV, potassium reversal potential *V*_*K*_ = −77 mV, leak reversal potential *V*_*l*_ = −54.4 mV. For the equations of the time-varying gate variables *m*, *h*, and *n* see Hansel et al. (1993). The injected constant currents (*I*_*i*_) are uniformly distributed random numbers (*I*_*i*_ ∈ [*I*_0_ − ε_*I*_, *I*_0_ + ε_*I*_], in this study *I*_0_ = 11.0 μA/cm^2^ and ε_*I*_ = 0.45 μA/cm^2^) and determine the intrinsic firing rate of the uncoupled neurons.

The coupling term *S*_*i*_ from Equation (1) (Popovych and Tass, 2012) contains a weighted ensemble average of all post-synaptic currents received by neuron *i* from the other neurons in the network and is given by:

(2) $$S_{i}=N^{-1}\sum_{j=1}^{N}\left(V_{r,j}-V_{i}\right)c_{ij}\left|M_{ij}\right|s_{j}.$$

*N* is the number of neurons within the ensemble, *V*_*r*, *j*_ is the reversal potential of the synaptic coupling (20 mV for excitatory and – 40 mV for inhibitory coupling), and *c*_*ij*_ is the synaptic coupling strength from neuron *j* to neuron *i*. There are no neuronal self-connections within the network (*c*_*ii*_ = 0 mS/cm^2^). *M*_*ij*_ has the form of a Mexican hat (Wilson and Cowan, 1973; Dominguez et al., 2006; De la Rocha et al., 2008) and defines the strength and type of neuronal interaction: strong short-range excitatory (*M*_*ij*_ > 0)and weak long-range inhibitory interactions (*M*_*ij*_ < 0). This spatial profile of coupling between neurons *i* and *j* is given by:

(3) $$M_{ij}=\left(1-d_{ij}^{2}/\sigma_{1}^{2}\right)\text{exp}\left(-d_{ij}^{2}/(2\sigma_{2}^{2})\right)$$

where *d*_*ij*_ = *d*|*i* − *j*| is the distance between neurons *i* and *j*,

(4) $$d=d_{0}/(N-1)$$

is the lattice distance between two neighboring neurons within the ensemble, *d*_0_ is the length of the neuronal chain, σ_1_ = 3.5, and σ_2_ = 2.0 as used in Popovych and Tass (2012). To minimize boundary effects, the neurons form a ring, which implies that *d*_*ij*_ = *d* · *min*(|*i* − *j*|, *N* − |*i* − *j*).

The synaptic variable *s*_*j*_ in Eqn. 2 is given by:

(5) $$\frac{ds_{j}}{dt}=\frac{0.5(1-s_{j})}{1+exp\left[-\left(V_{j}-5\right)/12\right]}-2s_{j}.$$

### Spike timing-dependent plasticity

In general, synaptic coupling strengths change depending on the precise timing of pre- and post-synaptic spikes (Markram et al., 1997; Bi and Poo, 1998). In the present study all synaptic weights *c*_*ij*_ were considered to be dynamic variables dependent on the time difference (Δ*t*_*ij*_) between the onset of the post- and pre-synaptic spikes *t*_*i*_, respectively *t*_*j*_ (Δ*t*_*ij*_ = *t*_*i*_ − *t*_*j*_). According to the spike timing-dependent plasticity (STDP) rule (Bi and Poo, 1998) the change in synaptic weight is given by:

(6) $$\Delta c_{ij}=\{\begin{array}{l} \beta_{1}e^{\frac{-\Delta t_{ij}}{\gamma_{1}\tau }}\;,\Delta t_{ij}\geq 0\\ \beta_{2}\frac{\Delta t_{ij}}{\tau }e^{\frac{\Delta t_{ij}}{\gamma_{2}\tau }}\;,\Delta t_{ij}<0 \end{array}$$

See Popovych and Tass (2012), In our model we update the synaptic weights *c*_*ij*_ in an event-based manner by adding δ · Δ*c*_*ij*_ for excitatory connections and −δ ·Δ*c*_*ij*_ for inhibitory connections with learning rate δ > 0 every time a neuron spikes. To avoid an unbounded strengthening or weakening, the synaptic weights are restricted to the interval *c*_*ij*_ ∈ [0, 1] mS/cm^2^ for excitatory synapses and *c*_*ij*_ ∈ [0, *c*_*max*_] mS/cm^2^ for inhibitory synapses with *c*_*max*_ = 1 unless stated otherwise. In this study the following values are used for the STDP parameters: β_1_ = 1, β_2_ = 16, γ_1_ = 0.12, γ_2_ = 0.15, τ = 14 ms, and δ = 0.002.

Due to STDP and the different intrinsic periods of the neurons, the synaptic weights change constantly. In this study the dynamics of the synaptic weights were investigated on a population level. The strength of the coupling within the neuronal population at time *t* is given by the synaptic weight averaged over the population:

(7) $$C_{av}\left(t\right)=N^{-2}\sum_{i,j}sgn\left(M_{ij}\right)c_{ij}\left(t\right),$$

with *M*_*ij*_ as defined in Equation (3) and the sign-function *sgn*. The amount of synchronization of the neuronal activity within the ensemble is influenced by the synaptic weights and can be represented by the order parameter (Haken, 1983; Kuramoto, 1984)

(8) $$R\left(t\right)=\left|N^{-1}\sum_{j}e^{i\varphi_{j}\left(t\right)}\right|,$$

Where φ_*j*_(*t*) = 2π(*t* − *t*_*j*, *m*_)/(*t*_*j*, *m* + 1_ − *t*_*j*, *m*_) for *t*_*j*, *m*_ ≤ *t* < *t*_*j*, *m* + 1_ is a linear approximation of the phase of neuron *j* between its *m*^*th*^ and (*m* + 1)^*th*^ spikes at spiking times *t*_*j*, *m*_ and *t*_*j*, *m* + 1_. The order parameter *R* measures the extent of phase synchronization in the neuronal ensemble and takes values between 0 (complete desynchronization) and 1 (perfect in-phase synchronization). For our data analysis the order parameter was averaged over the last 1.6 s of the CR-off period and will be denoted as average order parameter *R*_*av*_.

### Coordinated reset stimulation algorithms

Coordinated Reset (CR) stimulation was delivered to the neuronal ensemble of *N* spiking Hodgkin-Huxley neurons. This was done sequentially via *N*_*s*_ equidistantly spaced stimulation sites (Tass, 2003a): one stimulation site was active during *T*_*s*_/*N*_*s*_, while the other stimulation sites were inactive during that period. After that another stimulation site was active during the next *T*_*s*_/*N*_*s*_ period. All *N*_*s*_ stimulation sites were stimulated exactly once within one stimulation ON-cycle. Therefore, the duration of each ON-cycle is *T*_*s*_. This spatiotemporal activation of stimulation sites is represented by the indicator functions ρ_*k*_(*t*) (*k* ϵ {1, …, *N*}):

(9) $$\rho_{k}\left(t\right)=\{\begin{array}{ll} 1, & k^{th}\text{stimulation site is active at }t\\ 0, & \text{otherwise} \end{array}$$

The stimulation signals induced single brief excitatory post-synaptic currents. The evoked time-dependent normalized conductances of the post-synaptic membranes are represented by α-functions given by Popovych and Tass (2012):

(10) $$G_{stim}\left(t\right)=\frac{t-t_{k}}{\tau_{stim}}e^{-(t-t_{k})/\tau },\;t_{k}\leq t\leq t_{k+1}.$$

Here τ_*stim*_ = *T*_*s*_/(6*N*_*s*_) denotes the time-to-peak of *G*_*stim*_, and *t*_*k*_ is the onset of the *k*^*th*^ activation of the stimulation site. The spatial spread of the induced excitatory post-synaptic currents in the network is defined by a quadratic spatial decay profile (see Popovych and Tass, 2012 for motivation) given as a function of the difference in index of neuron *i* and the index *x*_*k*_ of the neuron at stimulation site *k*:

(11) $$D\left(i,x_{k}\right)=\frac{1}{1+d^{2}{\left(i-x_{k}\right)}^{2}/\sigma_{d}^{2}},$$

with *d* the lattice distance between two neighboring neurons as defined in Equation (4) and σ_*d*_ = 0.08*d*_0_ the spatial decay rate of the stimulation current.

The stimulation current from Equation (1) is given by:

(12) $$F_{i}=\left[V_{r}-V_{i}\left(t\right)\right]\;\cdot \;K\sum_{k\;=\;1}^{N_{s}}D\left(i,x_{k}\right)\rho_{k}\left(t\right)G_{stim}(t),$$

where *V*_*r*_ = 20 mV denotes the excitatory reverse potential, *V*_*i*_ the membrane potential of neuron *i*, K the stimulation intensity, and *D*, ρ, *G* are given by Equations (11), (10), and (9).

**Figure 1:**
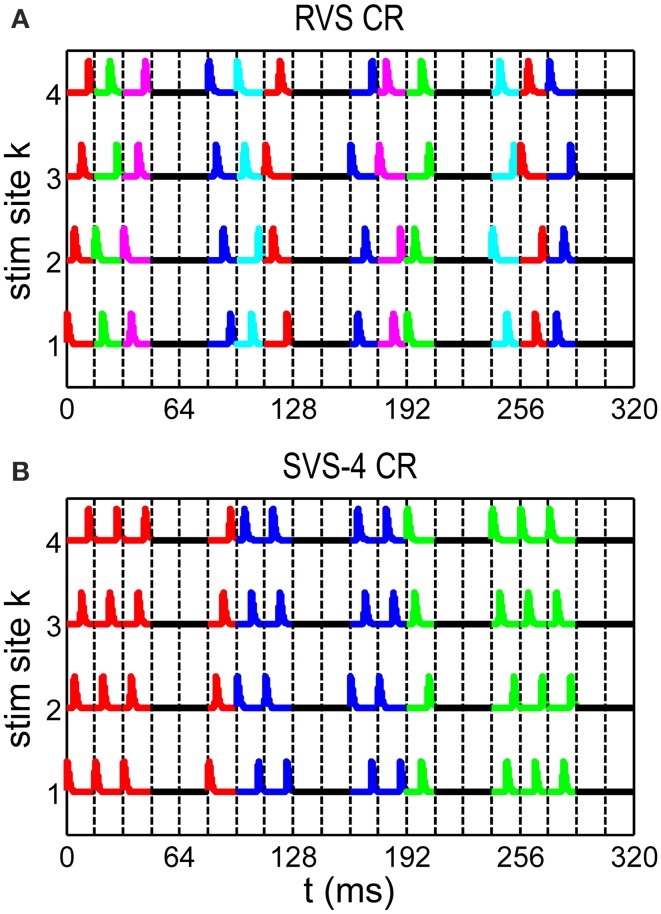
**Spatiotemporal stimulation signals of CR stimulation**. **(A)** An example sequence order for the rapidly varying sequences (RVS) CR. **(B)** An example sequence order for the slowly varying sequence CR with every sequence repeated 4 times (SVS-4) before the next sequence is used. A change of color indicates a new sequence. Vertical dashed lines separate stimulation ON- and OFF cycles: three ON-cycles are followed by two OFF-cycles.

In this paper we study three different CR algorithms: RVS CR stimulation (Tass and Hauptmann, 2006; Tass and Majtanik, 2006; Popovych and Tass, 2012; Tass and Popovych, 2012), FS CR stimulation (Tass, 2003a,b; Hauptmann and Tass, 2007, 2009; Tass and Hauptmann, 2007, 2009), and our novel SVS CR stimulation. During one sequence each stimulation site is activated exactly once. There are *N*_*s*_! (in our study 4! = 24) different sequences possible to stimulate *N*_*s*_ stimulation sites. In the RVS CR algorithm for each ON-cycle a new sequence was drawn randomly from the set of *N*_*s*_! possible sequences (see Figure 1A). For the slowly varying sequences CR algorithm (SVS-n) the sequence order is random and determined a priori in such a way that each sequence used, is consecutively repeated *n* times before another one is applied. For the SVS-4 CR stimulation signals as shown in Figure 1B one sequence was applied during the first *n* consecutive ON-cycles. After that the next sequence was applied during the next *n* consecutive ON-cycles, and so on (see Figure 1B for *n* = 4).

### Simulation details and data analysis

We ran simulations for different initial network conditions and different sequence orders. For each initial network condition the initial conditions of all *N* neurons were randomly drawn from uniform distributions (*n*_*i*_, *m*_*i*_, *h*_*i*_, *s*_*i*_ ∈ [0, 1]; *V*_*i*_ ∈ [−65, 5]*mV*; *I*_*i*_ ∈ [*I*_0_ − σ_*I*_, *I*_0_ + σ_*I*_]). The initial synaptic weights *c*_*ij*_ between the neurons were drawn from a normal distribution (*c*_*ij*_ ~ *N*(μ = 0.5 μ*A*/*cm*^2^, σ = 0.01 μ*A*/*cm*^2^)). After an initial equilibration phase of 2 s, STDP was included for the rest of the simulation. During the first 60 s with STDP the network was given the opportunity to rewire its connections without any influence from an external stimulation. At the end of this STDP-only period the network activity was highly synchronized and the CR simulation was applied for 64 s from *t* = 0 s on. During this CR-on period three stimulation ON-cycles alternated with two OFF-cycles as in the example stimulation signal shown in Figure 1. No stimulation was applied during the OFF-cycles. Each ON- and OFF-cycle lasted *T*_*s*_ = 16 ms. After 64 s the CR stimulation was stopped permanently and the 64 s lasting CR-off period started. After going through this procedure for one particular stimulation intensity, *K*, the procedure was repeated from *t* = 0 s on for the other *K*-values (*K* ∈ {0.10, 0.20, 0.30 …, 0.60}). For each CR stimulation this whole process was repeated for eleven different initial network conditions and sequence orders. Besides the RVS CR stimulation also the SVS-100 CR stimulations were applied for eleven different combinations of initial conditions and sequence orders. Finally, the optimal number of different sequences used in the SVS CR stimulation was explored.

The resulting values of *C*_*av*_ (Equation 7) at *t* = 128 s and *R*_*av*_ (Equation 8) averaged over the last 1.6 s of the CR-off period) were plotted in boxplots (Tukey, 1977). In order to compare the results of different CR algorithms for a constant stimulation strength, *K*, the obtained boxplots are plotted next to each other, whereby the color represents which CR algorithm was used. Statistical significances of differences between the results of the different CR algorithms were determined by the one-sided Mann-Whitney test.

## Results

### Slowly varying sequences boost CR stimulation effect

To verify whether the SVS CR stimulation is more successful than the RVS CR stimulation, the effect of both CR algorithms on the average synaptic weight, *C*_*av*_, as well as on the synchronization of neuronal activity *R* has to be investigated. Each measure will be explored first for the RVS and then for the SVS CR stimulation.

**Figure 2:**
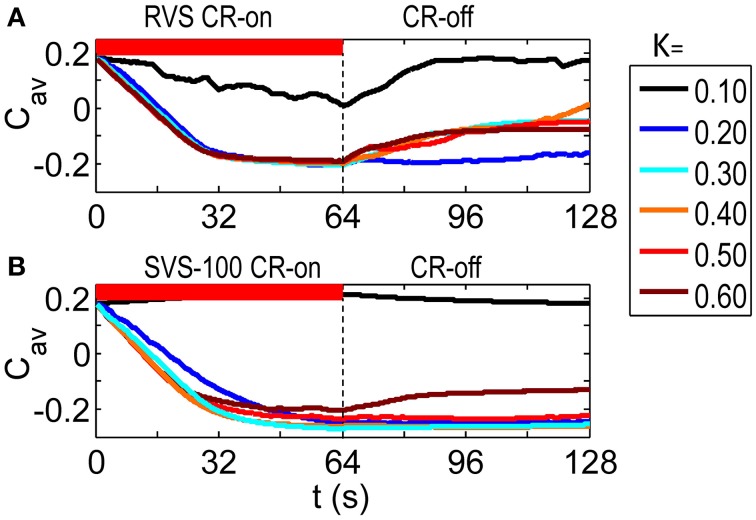
**Dynamics of the average synaptic weight, *C*_*av*_, for different stimulation intensities, *K***. **(A)** Results of the RVS CR stimulation. **(B)** Results of the SVS-100 CR stimulation. The initial network is the same for all simulations. The sequence order used for each CR method is constant for all *K*-values. The CR-on period, represented by the red horizontal bar, starts at *t* = 0 s and is switched off at *t* = 64 s (dashed vertical line). During the subsequent 64 s CR-off period, no stimulation is delivered, and *C_*av*_* evolves spontaneously. *c*_*max*_ = 1 for all simulations.

As visualized in Figure 2A the RVS CR stimulation causes a weakening of the average synaptic weight *C*_*av*_ during the CR-on period for all stimulation intensities *K*. At the end of the subsequent CR-off period, the average synaptic weight is still much weaker than before the CR stimulation was applied, except for the weakest stimulation intensity. Figure 2B then shows how the SVS CR stimulation, delivered to the same initial network, decreases the average synaptic weights even more and causes in general lower long-lasting *C*_*av*_–values compared to the RVS CR stimulation. Since we are interested in the long-lasting effects of the CR stimulation period, we will concentrate on the values at the end of the CR-off for the remainder of this work.

**Figure 3:**
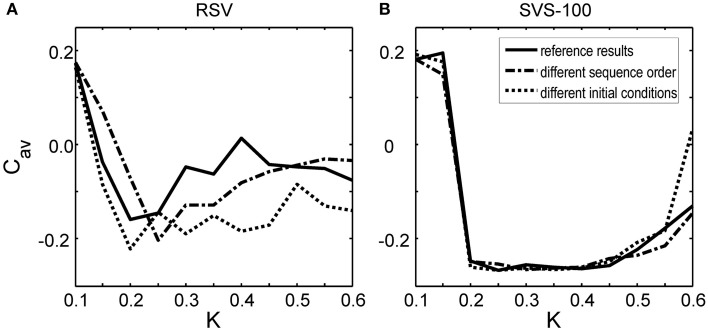
**Effect of the sequence order and of the initial network conditions on the average synaptic weight *C*_*av*_ at *t* = 128 s as a function of stimulation intensity *K***. **(A)** *C*_*av*_-values at *t* = 128 s obtained by the RVS CR stimulation. **(B)** *C*_*av*_-values at *t* = 128 s obtained by the SVS-100 CR stimulation. The *C*_*av*_(*t* = 128 s) values in Figure 2 are the reference results and represented by the solid lines in this Figure. The dashed-dotted lines show the result for a simulation with the same initial network conditions as used to obtain the reference results but for another randomly chosen sequence order. The dotted lines represent the obtained *C*_*av*_-values at *t* = 128 s for a simulation with the same sequence order as used to obtain the reference results, but for other initial network conditions.

To investigate whether this observed improvement by the SVS CR stimulation is just a coincidence, we have also changed the sequence order or the initial network conditions. Figure 3A shows that by applying another RVS order to the same initial network or by applying the initial sequence order to a network with different initial conditions, different long-lasting *C_*av*_*-values were obtained. Only for the weakest stimulation intensity, *K* = 0.10, the RVS algorithm caused similar long-lasting *C*_*av*_-values. For other stimulation intensities, it suggests that the effect of the RVS CR stimulation depends on the sequence order used and on the initial network conditions. As follows from Figure 3B, the success of the SVS-100 CR stimulation depends less strongly on the exact sequence order and the initial network conditions and the SVS-100 CR stimulation results in a smaller *C*_*av*_-value than the RVS CR stimulation, over a wide range of stimulation intensities *K* continuing the superiority of this method.

**Figure 4:**
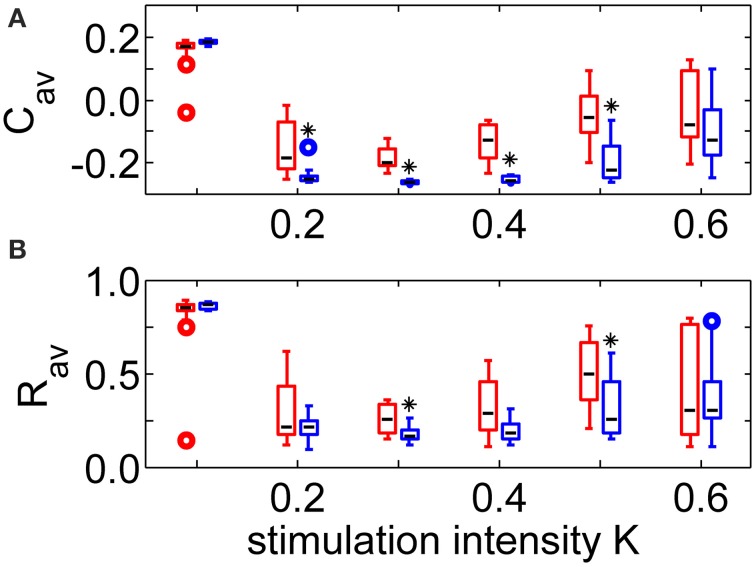
**Comparison of the anti-kindling effects at *t* = 128 s for the RVS and the SVS-100 CR stimulation**. **(A)** Boxplots of the average synaptic strength, *C*_*av*_, at *t* = 128 s as a function of the stimulation intensity, *K*, for the RVS and the SVS-100 CR stimulation. **(B)** Boxplots of the order parameter *R* averaged over the last 1.6 s, *R*_*av*_, as a function of *K* for the RVS and the SVS-100 CR stimulation. The RVS CR results for the same *K*-values are shown in red and slightly shifted to the left and the SVS-100 results are shown in blue and slightly shifted to the right. The black lines within the boxes show the medians for each condition, the boxes the middle 50% and the whiskers below (above) the boxes the first (last, respectively) 25%. Outliers are defined as 1.5 times the length of the box below or above the box and represented by open circles. For each condition (*K*-value and type of CR) the simulations are repeated eleven times for different initial conditions of the network in combination with different sequence orders. One asterisk indicates a significantly lower *C*_*av*_ - or *R*_*av*_-value compared to the values obtained by the RVS CR stimulation (one-sided Mann-Whitney test with *p* < 0.05).

Robustness against variations of the sequence order and against initial network conditions is of crucial importance for the CR therapy. Therefore, all stimulations were repeated 11 times for different combinations of initial network conditions and sequence orders. The boxplots in Figure 4A show that the long-lasting effect of decreasing the average synaptic strength is significantly better for the SVS-100 than for the RVS CR stimulation over a wide range of stimulation intensities *K* (one-sided Mann-Whitney test, *p* < 0.05). Besides generating a better *C_*av*_*-value, the SVS CR stimulation is also more robust against initial network conditions and sequence orders. The SVS-100 also induces a significant smaller *R*_*av*_ than the RVS CR stimulation (one-sided Mann-Whitney test *p* < 0.01), but for a smaller set of *K*-values as shown in Figure 4B. *R*_*av*_ is the value of the order parameter averaged over the last 1.6 s of the CR-off period.

To rule out false estimates of the time averaged order parameter *R, we* used different window lengths. False estimates could, for instance, be caused by low-frequency oscillations of *R* with periods exceeding the window length used for our averaging analysis. In our analysis of the order parameter *R*, presented in this paper, we averaged over the last 1.6 s of the 64 s during CR-off period. Averaging *R* over a quarter (=16 s) of the total CR-off period gave very similar results. Hence, we can consider our results to be sufficiently robust with respect to variations of the length of the time window used for our evaluation.

Simulations for SVS-25 CR stimulation during the 64 s lasting CR-on period gave similar results for *C*_*av*_ and *R*_*av*_ as the SVS-100 CR (results not shown), illustrating that 25 consecutive repetitions of each sequence are already enough to improve the CR effect.

**Figure 5:**
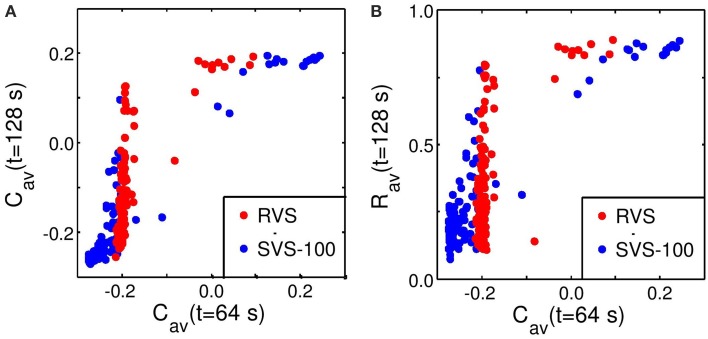
**Predictability of the anti-kindling effect by C_*av*_ at *t* = 64 s**. **(A)** Relation between *C*_*av*_ at the end of the CR-on period (*t* = 64 s) and at the end of the CR-off period (*t* = 128 s). **(B)** Relation between *C*_*av*_ at the end of the CR-on period (*t* = 64 s) and *R*_*av*_ at the end of the CR-off period (*t* = 128 s). The *C*_*av*_-values at *t* = 64 s are calculated and plotted against *C*_*av*_ or *R*_*av*_ at *t* = 128 s for each condition (initial network conditions, stimulation intensity, sequence order, *c*_*max*_ = 1) as used for Figure 4. Red circles represent the results of the RVS CR stimulation, blue circles of the SVS-100 CR stimulation. Note that the red circles are plotted on top of the blue circles.

From the standpoint of clinical applications it is important to understand the relationship between the acute effect achieved during stimulation and the after-effect observed after cessation of stimulation. To this end, we studied the relationship between the values of *C*_*av*_ and *R*_*av*_ at the end of the CR-on period (*t* = 64 s) and their values at the end of the CR-off period (*t* = 128 s) (Figure 5). The relation between *C*_*av*_ at *t* = 64 s and at *t* = 128 s is visualized in Figure 5A. In a first approximation, small values *C*_*av*_ and *R*_*av*_ at *t* = 64 s are required but not necessarily sufficient for small values of *C*_*av*_ and *R*_*av*_ at *t* = 128 s. Hence, with a certain probability a pronounced acute stimulation effect is associated with a good long-term outcome. In contrast, poor acute stimulation effects are not related to pronounced after-effects.

### Optimal number of different sequences used for SVS CR stimulation

In this section we analyze the impact of sequence changes on the dynamics of the average synaptic connectivity as assessed by *C*_*av*_. To this end, first, we perform a CR stimulation with fixed sequence (FS CR) and compare it to CR stimulation epochs where the sequence is either changed once or at three equidistant times without changing the total duration of the CR-on period. This implies that the number of different sequences multiplied with the number of consecutive repetitions, *n*, is constant. Finally, the optimal number of different sequences used in the SVS CR stimulation was explored.

**Figure 6:**
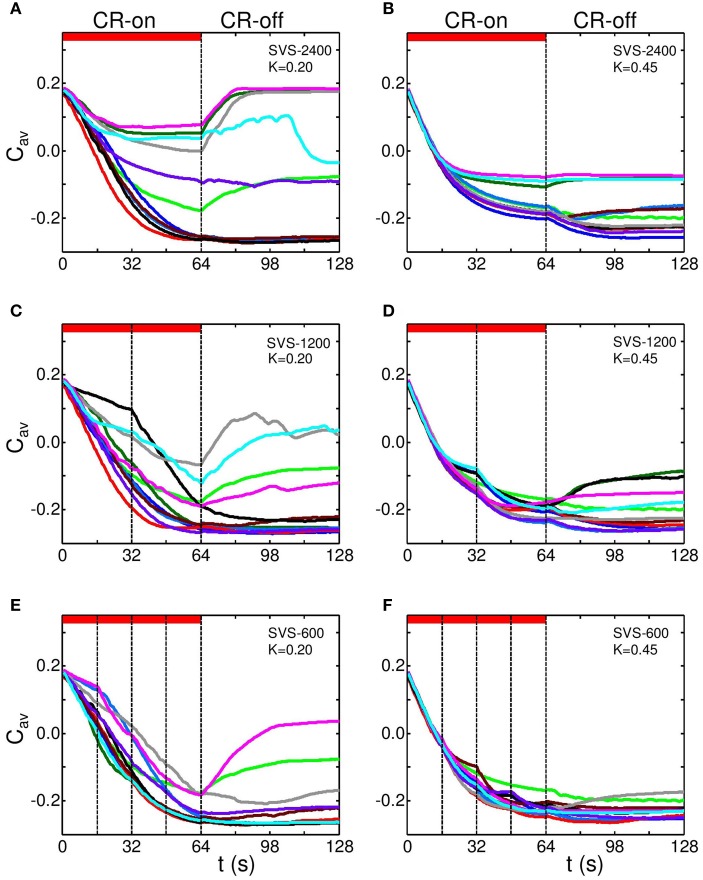
**Effect of switching the sequence during CR stimulation**. In a series of simulations with different stimulation intensities (*K* = 0.20 in left panels and *K* = 0.45 in right panels) the sequence was either kept fixed [FS CR stimulation, **(A,B)**], randomly varied just once [in the middle of the stimulation period, at *t* = 32 s, **(C,D)**] or randomly varied at three equidistant times [at *t* = 16, 32, 48 s, **(E,F)**]. Simulations were performed for eleven different sequence orders and initial network conditions. Each panel shows the dynamics of *C*_*av*_ for each of the eleven simulations in a different color. FS CR stimulation **(A,B)**: Time course of *C*_*av*_ for eleven combinations of different initial network conditions and different sequence for SVS-2400, respectively, for *K* = 0.20 **(A)** and *K* = 0.45 **(B)**. Change of sequence in the middle of the stimulation epoch **(C,D)**: Two different sequences, each applied 1200 times in a row. Change of sequence at *t* = 32 s, with *K* = 0.20 **(C)** and *K* = 0.45 **(D)**. Sequence is changed three times at equidistant times (*t* =, 16, 32, and 48 s) during the stimulation epoch **(E,F)**: In each simulation four different sequences are applied 600 times in a row, so that after 16 s the next sequence randomly chosen, with *K* = 0.20 **(E)** and *K* = 0.45 **(F)**. The red horizontal bars represent CR-on periods. The vertical dashed-dotted lines indicate a change of sequence. *c*_*max*_ = 1 in all simulations.

We analyzed the effect of *FS CR stimulation* for eleven different initial network conditions in combination with a different sequence for each network, respectively. Figure 6A clearly shows that for the FS CR stimulation (SVS-2400) with *K* = 0.20, the decrease of *C*_*av*_ strongly depends on which sequence is used. Pronounced long-lasting effects are achieved by some sequences, whereas no anti-kindling is observed for other sequences. Increasing the stimulation intensity to *K* = 0.45 improves the robustness of FS against the choice of the sequence used and the initial network conditions (Figure 6B). For *K* = 0.45 the average synaptic weight stabilizes at a small to intermediate value, depending on the sequence and the initial network conditions. The stabilization of *C*_*av*_ is more rapidly achieved at higher stimulation intensity *K*.

By using *two different sequences* instead of just one sequence, the first sequence may stabilize *C_*av*_* at an intermediate value of *C*_*av*_ and, hence, lead to a sub-optimal outcome. However, at *t* = 32 s the second sequence takes over, and may further reduce *C*_*av*_ as shown by its kinks at *t* = 32 s (Figure 6C for *K* = 0.20 and SVS-1200), in particular, for the more effective stimulation intensity *K* = 0.45 (Figure 6D).

By the same token, the long-lasting effects on the mean synaptic connectivity *C*_*av*_ and the robustness of the stimulation further improve by using *four different sequences* (SVS-600, Figure 6E for *K* = 0.20 and Figure 6F for *K* = 0.45). Again, especially at higher stimulation intensity changes of the sequence may come with a stepwise-like further reduction of *C*_*av*_ showing up as kinks in the time course of *C*_*av*_ at times when sequences are changed (*t* = 16, 32, and 48 s).

**Figure 7:**
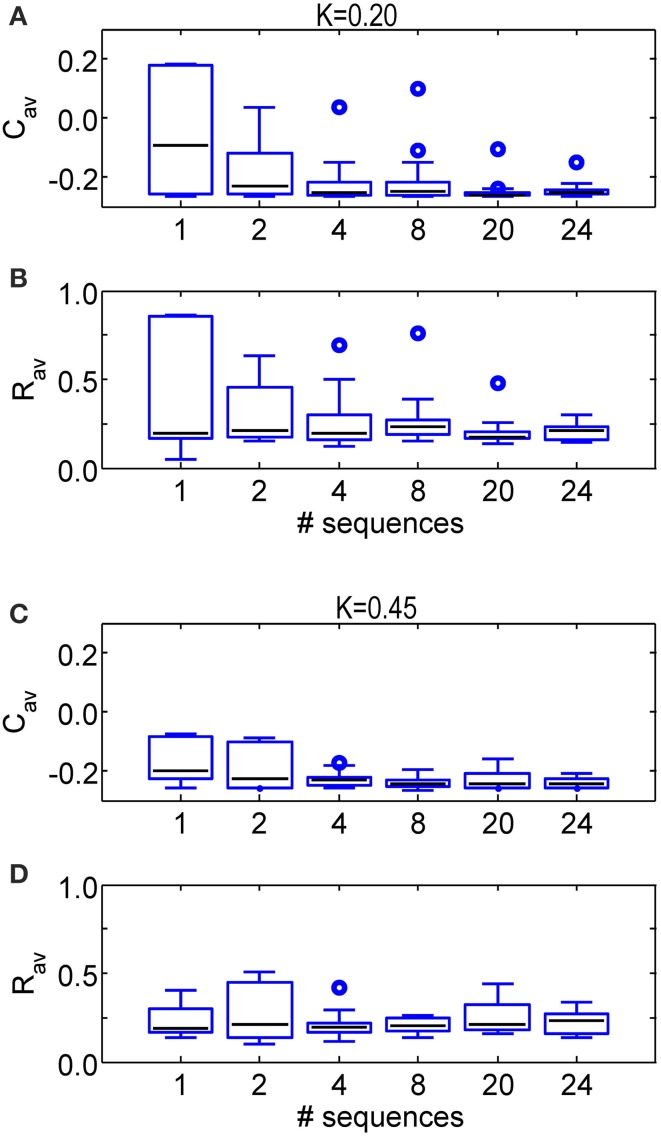
**Comparison of the anti-kindling effects for some numbers of different sequences applied during the SVS CR stimulation**. **(A)** Boxplots of *C*_*av*_ at *t* = 128 s for different numbers of sequence changes used in the SVS CR stimulation with *K* = 0.20. **(B)** Boxplots of *R*_*av*_ at *t* = 128 s for some numbers of different sequences used in the SVS CR stimulation with *K* = 0.20. **(C)** As in **(A)** for *K* = 0.45. **(D)** As in **(B)** for *K* = 0.45. The black lines within the boxes show the medians for each condition, the boxes the middle 50% and the whiskers below (above) the boxes the first (last, respectively) 25%. Outliers are defined as 1.5 times the length of the box below or above the box and represented by open circles. For each condition (*K*-value and number of sequences) the simulations are repeated eleven times for different initial conditions of the network in combination with different sequence(s). The number of consecutive sequence repetitions was adjusted with respect to the number of different sequences so that the duration of the CR-on period is always 64 s for each simulation. For example if two different sequences are used, each of them is repeated 1200 times in a row, in case four different sequences are used, each of them is repeated 600 times.

Analogously, we further increase the number of different sequences used during one CR epoch. Figure 7 shows the stimulation outcome in terms of synaptic connectivity *C*_*av*_ (Figures 7A,C) and order parameter *R*_*av*_ (Figures 7B,D) averaged over the last 1.6 s of the CR-off period for different stimulation intensities (*K* = 0.20 in Figures 7A,B and *K* = 0.45 in Figures 7C,D). The statistics obtained from a set of eleven simulations performed for different initial network conditions and sequence orders shows that the main part of the SVS-induced improvement of the CR effect is already achieved with four different sequences. Using more than four different sequences hardly leads to a further reduction of *R*_*av*_ and *C*_*av*_ and their variability.

## Discussion

Our results show that the SVS CR stimulation leads to significantly weaker average synaptic weights than the RVS CR stimulation over a wide range of stimulation. Within this range the Inter-Quartile-Range (25th to 75th percentile) is smaller for the SVS CR approach compared to the RVS CR. This implies that the SVS CR approach is more robust against initial conditions of the network and against the order of the sequences than the RVS CR in this range. The differences between the results of the SVS with 25 and 100 consecutive repetitions of each sequence are in general not significant, although more repetitions tend to have a larger impact on the average synaptic weight (results not shown). A more significantly reduced average synaptic weight does not necessarily translate into more significantly reduced overall synchrony. In fact, for the SVS CR stimulation the network activity was significantly more desynchronized than for the RVS CR stimulation in a smaller range of stimulation intensities than for the weakening of the network connectivity.

Optimal anti-kindling is obtained at intermediate stimulation intensities (Figures 3, 4). This is in agreement with previous computational studies (e.g., Lysyansky et al., 2011a; Popovych and Tass, 2012; Ebert et al., 2014). On the one hand CR stimulation has to be of sufficient intensity to achieve phase resets of the different subpopulations, but on the other hand at high intensities the subpopulations are no longer separately stimulated. In the limiting case of very high intensities each stimulus affects nearly the whole neuronal population and causes an entrainment of the whole population which fosters synchronization rather than desynchronization. Our results are stable with respect to variations of model parameters, e.g., by doubling the maximum allowed inhibitory synaptic weight (*c*_*max*_ = 2 for inhibitory synapses, results not shown).

Our results show that optimal long-lasting desynchronization requires the right combination of appropriate repetition and occasional variation of sequences. In fact, SVS-CR stimulation is better than FS CR stimulation over a wide range of stimulation intensities. Furthermore, the optimal number of different sequences for the SVS CR stimulation is four or more. This implies that repetition alone, like in the case of FS CR simulation (Hauptmann and Tass, 2007, 2009; Tass and Hauptmann, 2007, 2009; Tass et al., 2009), is not the only ingredient for the improvement of CR stimulation. With insufficiently many different sequences in the SVS CR approach, the network can stabilize in a local minimum that is much larger than the global minimum *C*_*av*_-value for a given stimulation intensity. In case the sequence is replaced after many repetitions by another sequence and again after a large number of repetitions by another sequence, the network connectivity can stepwise decrease from one local minimum to another, in this way, approaching the global minimum for a given stimulation intensity. Using more than four different sequences in the SVS CR stimulation does not significantly improve the long-lasting anti-kindling effects compared to those obtained with just four different sequences. The different local minima correspond to different attractors of the network (see Popovych et al., 2015). In fact, in our model network a multitude of attractors with different amount of mean synaptic weight and neuronal synchrony coexist, covering the whole spectrum from minimal mean connectivity and synchrony up to strongly up-regulated mean connectivity and synchrony. Hence, our results indicate that SVS CR stimulation prevents the network from getting stuck in undesirable attractors (with intermediate mean connectivity and synchrony) in the course of the anti-kindling stimulation.

Another difference between the SVS-100 and the RVS CR stimulation is that for the SVS CR stimulation by definition in a suitably large time window each sequence is repeated exactly 100 times, but that for the RVS CR stimulation the number of (timely separated) repetitions of each sequence can vary within such a time window, since by definition the sequence of the RVS CR changes from ON-cycle to ON-cycle, where each sequence occurs with equal probability. For an infinitely long time window also for the RVS CR stimulation the different sequences will occur with equal probability. However, on the time scale of one completed series of sequences of the SVS-100 CR stimulation, i.e., for larger, but not infinitely large numbers of sequences, this may be different. Taking a permutation of all 2400 applied sequences (including the repeated sequences) of a SVS-100 CR stimulation generates a CR stimulation signal in which the different sequences occur randomly, but each still exactly 100 times. Simulations with this permutated CR stimulation signal show that *C*_*av*_ and *R*_*av*_-values are similar to those obtained by the RVS CR stimulation although the spread is in general larger for the permutated than the random signal (results now shown). This suggests that a constant frequency with which each sequence occurs in a wider time window does not contribute to the success of the SVS CR stimulation, but that it is mainly determined by the consecutive repetitions of a sequence and the number of different sequences. This is actually supported by the fact that already four different sequences in the SVS CR stimulation are sufficient to induce a full-blown anti-kindling (see above).

Applying our SVS CR approach to DBS may be particularly rewarding, since with the same stimulation intensity as used for RVS CR or fixed sequence CR, SVS CR might lead to a better therapeutic outcome. Reducing the stimulation energy will likely lead to a reduction of the rate of side effects. RVS CR DBS was successfully applied at stimulation amplitudes (of the single stimulation pulses) similar to those of standard permanent high-frequency DBS (Adamchic et al., 2014a) as well as corresponding to a third of the amplitude used for standard permanent high-frequency DBS (Tass et al., 2012a). Accordingly, within that range of stimulation amplitudes SVS CR-DBS might be superior to RVS CR-DBS. However, given the intensity dependence of the anti-kindling effects (e.g., Figure 4), systematic dose finding studies for both types of CR-DBS are required to best exploit their actual clinical potential. By the same token, systematic dose finding studies should be conducted for acoustic CR stimulation for the treatment of tinnitus (Tass et al., 2012b) for SVS CR. As yet, acoustic RVS CR stimulation was delivered at only one stimulation intensity (i.e., loudness level), namely for just super threshold loudness. In the context of dose finding studies the results from Figure 5 might be important, since they show that—at least in the model under study—acute effects (achieved during stimulation) are necessary but not sufficient for pronounced long-term desynchronization effects observed after cessation of stimulation.

In a previous computational study it was shown that FS CR stimulation may augment brain function by counteracting cerebral hypo-activity without promoting pathological neuronal synchrony (Lysyansky et al., 2011b). Accordingly, a forthcoming study might focus on the comparison of the potential of SVS CR for activating brain areas and protecting the brain from abnormal synchrony and kindling as opposed to both FS CR and RVS CR.

### Conflict of interest statement

Peter Tass is employed by Jülich Research Center and works as consulting professor at Stanford University. Formerly working with ANM GmbH (Cologne, Germany) and shareholder of ANM GmbH. Several patents protect invasive and non-invasive CR neuromodulation stimulation. The main inventor of the CR patent portfolio is Peter Tass, the assignee is Jülich Research Center. Magteld Zeitler is working at Jülich Research Center. Peter Tass and Magteld Zeitler are co-inventors of recently filed SVS CR patents.
